# Insights on the Impacts of Accelerometer Location on the Dynamics and Characteristics of Complex Structures

**DOI:** 10.3390/s23249830

**Published:** 2023-12-14

**Authors:** Adam Takeshita, Jonah Madrid, Ezekiel Granillo, Abdessattar Abdelkefi

**Affiliations:** Department of Mechanical and Aerospace Engineering, New Mexico State University, Las Cruces, NM 88003, USA; adamrt@nmsu.edu (A.T.); jonahmad@nmsu.edu (J.M.); zg2019@nmsu.edu (E.G.)

**Keywords:** complex systems, dynamics environmental testing, modal analysis, experimental measurements, accelerometer placement, nonlinear dynamics

## Abstract

There exists a high level of difficulty in understanding the physical responses of complex dynamical systems. To this end, researchers have previously used different measurement techniques, such as displacement sensors or accelerometers, in the laboratory to capture a system’s dynamics. A well-known structure in the literature is the Box Assembly with Removable Component (BARC) whose purpose is to gain a deep understanding of testing complex systems. Further breaking down the structure, the Removable Component (RC) portion is often used as a relatively simplified version which retains significant complexity from the original system. However, the placement of accelerometers on the RC have varied greatly throughout the literature which presents a challenge in comparing results and may not accurately represent the system’s dynamics. Finite Element Analysis (FEA) is performed for three common accelerometer locations to determine how their placement affects the frequencies and mode shapes for the RC and results are compared against those without accelerometers. Free vibration experiments are carried out to understand the variation of frequencies and damping for each accelerometer location to obtain the overall response for the first mode of vibration. Next, random vibration experiments are run to gain insight on the interaction between linear and nonlinear responses based on excitation level, while showing the influence of an accelerometer’s location on system dynamics. The results demonstrate that the location of the accelerometer is highly influential on the linear and nonlinear characteristics of the system. It is proved that for the first mode of vibration, nonlinear softening and nonlinear damping behaviors may take place due to the interaction between the location of accelerometers, direction of excitation, and response axis analyzed.

## 1. Introduction

Vibration testing is one of the most popular methods of experimentation used to understand complicated systems and their dynamical responses. It is often used for systems such as weapons, pipelines, turbines, aircraft, or nuclear plants that exhibit high complexity as vibration testing is often a significantly cheaper alternative than subjecting the system of interest to its service environment [1,2,3,4]. Failure to understand the dynamical responses for such complex systems may lead to unexpected costs, delays, or even catastrophic failure. Due to the prohibitive costs of testing these complex structures, the problem may be further broken down in a process known as substructuring, which aims to divide the system into smaller components and gage their individual responses. The intention is that by analyzing smaller portions of interest, a deeper understanding may be gained about the system. One common method is known as frequency-based substructuring, which aims to relate dynamical systems to each other by Power Spectral Densities (PSDs) and has been widely investigated [5,6,7]. Another popular model of substructuring was introduced by Craig-Bampton, which utilizes Finite Element Analysis (FEA) to truncate a system of equations through a combination of structure modes and boundary points [8,9,10].

While substructuring can be an efficient approach, two issues arise. First, FEA models are notoriously difficult to match to physical systems for a myriad of reasons from mesh generation to computational costs and yield an extensively idealized representation of the system, though the component itself behaves in a nonideal manner [11]. Second, by nature substructuring inherently alters the system, creating room for discrepancies in experimental data by analyzing lighter systems, which are more effected by laboratory instrumentation.

To further investigate substructuring methods, researchers at Sandia National Laboratories and Kansas City National Security Campus introduced a challenge problem to the literature known as the Box Assembly with Removable Component (BARC) [12,13]. The BARC was created as a test fixture to investigate boundary conditions and fixture effects on a system’s dynamics in a simplified model while still retaining a high level of complexity. The Removable Component (RC) portion of the BARC acts as a substructured unit under test and the Box Assembly (BA) represents a non-traditional flexible fixture in an attempt to match a possible service environment. Because the BARC has multiple bolted joints, it serves as a complex nonlinear system in which small changes in boundary conditions or laboratory instrumentation may have significant impacts on its dynamical responses. Researchers have heavily studied the BARC system since its inception and many variations in experimental setup exist in the literature, one of the most notable being the variations in boundary conditions between the BARC and shaker [14,15,16,17]. These variations present a challenge for researchers to compare results as shown in [18] where the authors proved that decreasing the distance between bolted joints connecting the box assembly significantly decreases the resulting natural frequencies of the system and may cause contact nonlinearities with the fixture.

Due to the BARC system’s complexity, many studies have further broken down the problem by replacing the box assembly or by focusing only on the RC, which retains numerous bolted joints and still provides a challenging system. Throughout the literature, much emphasis has been placed on matching laboratory tests to service environments even for the RC. Ideally, empirical data are collected from a system-level test and used to design laboratory testing at the interface between the component of interest and fixture. However, a known fault of this method is that designing fixture dynamics to match those of the integrated interface is exceedingly difficult. This problem is commonly described as impedance mismatch to which much effort has been devoted [12]. The authors in [19,20,21,22] created a new fixture for testing by replacing the box assembly to investigate impedance matching, a novel topic that attempts to shift the paradigm in random vibration testing. Preliminary results showed that impedance matching may yield a promising method for active vibration control and that the changed boundary conditions can have significant impacts on the system. Others like Paripovic et al. [23] have investigated up to six-degrees of freedom (DOF) tests to better replicate service environments for the RC as laboratory tests often utilize only a single axis. This test reinforces the importance of replicating service environments through multi-axis testing. Similarly, Napolitano et al. [24] varied excitation location and investigated more six-DOF tests by instead attaching shakers to the RC’s c-channels.

Taking DOF tests one step further, Skousen et al. [25] and Schoenherr et al. [26] doubled the total shaker inputs to create a 12-DOF environment to obtain modal parameters. By subjecting the RC to a more realistic environment, results from these studies led to an improved correspondence between the service environment and laboratory. Moving in a different direction, Hall [27] sought to minimize the difference in environments in an “N + 1” style of fixture design by varying the fixture dimensions for the RC. This method provides alternative options to elicit desired responses from the fixture. While single-axis tests do not typically represent a realistic case, multi-axis testing may not always be feasible for certain systems or laboratories. Smith and Brake [16] offered a hybrid method for axis variation by investigating single-axis excitation in multiple axes and using superposition to combine responses and gain a deeper understanding of the system’s dynamics. Overall, previous studies on the RC have focused on fixture design or methods of excitation to bridge the gap between laboratory and service environments.

The research studies in the literature have innovatively guided researchers to conduct increasingly accurate laboratory tests but have typically neglected impacts of the instrumentation on the system’s dynamics. Two of the most popular methods for instrumentation include Laser Doppler Vibrometers (LDVs) and tri-axial accelerometers [28]. LDVs have been introduced as an alternative to classical accelerometers but have several issues. For example, in general, LDVs struggle to measure large displacements, have lengthy acquisition times, are more difficult to set up, and are costly [29,30,31,32]. Additionally, in the service environment, LDVs are typically not used as they are far bulkier than accelerometers and can be affected by vibration where they are attached to the system [33]. Rossi [34] has also pointed out that LDVs are limited to measuring relative values, while accelerometers are able to measure absolute displacement. For these reasons, accelerometers are often the sensors of choice for investigating the dynamical responses of complex systems. Accelerometers, however, come with their own unique tradeoffs. Accelerometers add mass to the component being tested in a phenomenon known as “mass loading effect” [35,36,37,38,39,40]. The added mass may contribute unwanted dynamics to the system, but if understood, can be adequately negated or corrected.

To ensure that the laboratory tests match the environmental conditions of the fully integrated system, the impacts of the accelerometer’s placement on the dynamics of complex structures, or RC in this study, are evaluated to determine the possible issues and challenges. To this end, the goals of this study are twofold: first to provide a deeper understanding of how accelerometer’s placement impacts the resonant frequencies and linear/nonlinear damping of the RC and second, to determine locations that minimize their contribution to the RC’s dynamics. To this extent, three accelerometer’s placements on the RC commonly found in the literature are explored and analyzed through FEA and two types of vibratory excitations. A description of the system is introduced in Section 2, along with FEA and experimental setups. In Section 3, linear characteristics of the system are investigated through FEA with a focus on the mode shapes and natural frequencies based on the accelerometer’s placement. The experimental results from free and random excitations are compared in Section 4 for the resonant frequencies and damping characteristics in both linear and nonlinear regimes. Finally, conclusions from the work are presented in Section 5.

## 2. System’s Description and Experimental Setup

**Figure 1:**
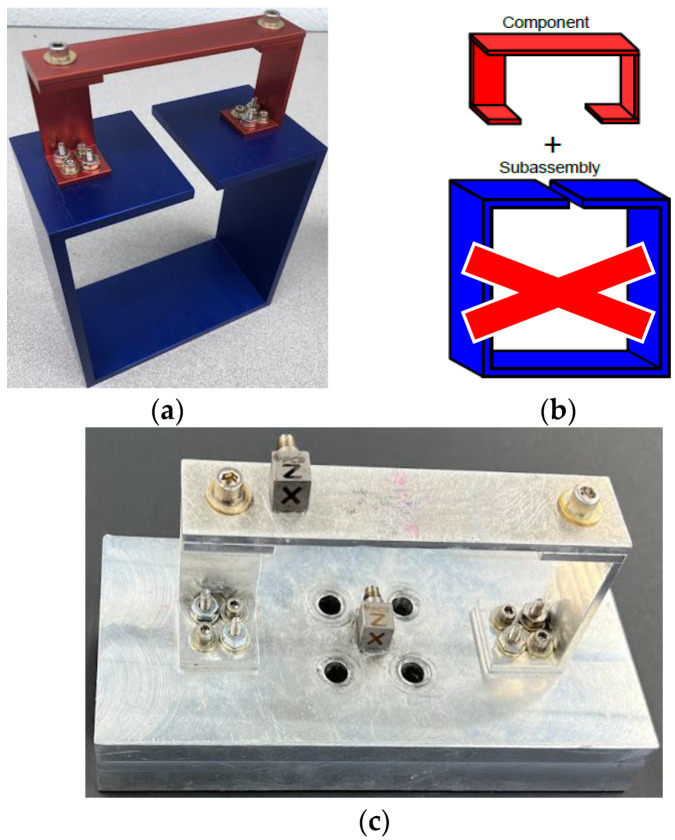
(**a**) A physical BARC system, (**b**) BARC schematic, and (**c**) physical RC.

Pictures of the entire BARC system are given in Figure 1a,b in addition to the RC in Figure 1c. Due to the complexity of the system, this study focuses on the RC portion of the BARC system which is still rich in terms of dynamics and coupling. The RC’s dimensions are outlined in the challenge problem designed by Sandia National Laboratories and Kansas City National Security Campus [13,41]. The RC weighs 91.8 g and consists of two c-channels made from aluminum 6063 with a beam atop made from aluminum 6061. The fixture used is made from another aluminum alloy with sufficient thickness to negate its influence on the dynamics of the RC. Bolts are torqued in accordance with the challenge problem specifications and while some researchers have added adhesives to the joints to minimize nonlinearities, none are used in this study.

**Figure 2:**
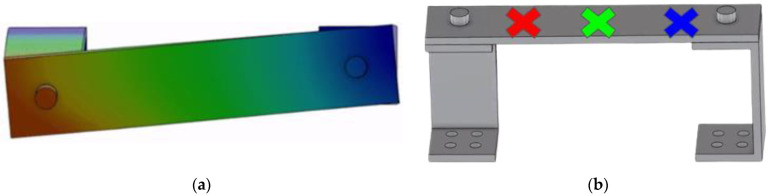
FEA of the RC: (**a**) von Mises plot and (**b**) left (red), middle (green), and right (blue) accelerometer’s locations.

To gain a fundamental understanding of the system, the RC is first investigated through Finite Element Analysis (FEA) using ABAQUS commercial software (3DEXPERIENCE 2022). Computational simulations are performed through the frequency step analysis function to determine mesh convergence, followed by natural frequencies and mode shapes of the system. Figure 2 demonstrates an example of these visuals using a von Mises yield stress method to demonstrate displacement. Accelerometer locations are chosen from popular locations in the literature that correspond to approximate nodes and anti-nodes or largest displacement areas in the RC’s first mode of vibration. This is specifically to compare the results that are obtained from all locations and the effects that the accelerometer has on the calculated values for the natural frequencies and displacements. For example, in several previous studies [14,15,16,42,43], the accelerometers are often seen in the middle of the beam connecting the two c-channels on the Removable Component, or near the top bolted connections. These observations, with a particular focus on the first mode of vibration, lead to the conclusion of testing the three locations shown in Figure 2. Accelerometers weighing 6.3 g are placed on the top plate of the RC 2.54 cm from the left edge, at the center of the plate, and 2.54 cm from the right edge, representing the left, middle, and right locations, respectively. Throughout this study, when referring to the placement, the left accelerometer location is in red, the middle accelerometer is represented in green, and the right accelerometer is in blue, as depicted in Figure 2.

**Figure 3:**
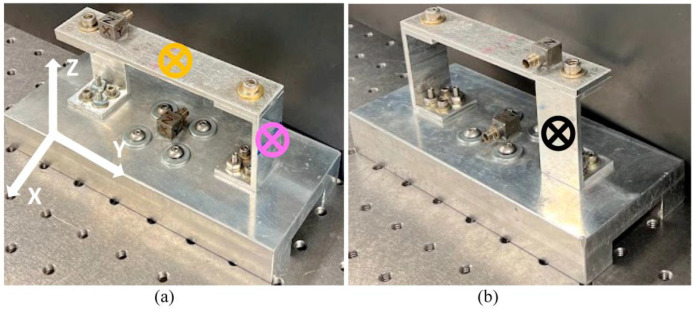
Free vibration impact location in *X*-axis (black), *Y*-axis (magenta), and *Z*-axis (orange) for (**a**) front and (**b**) back views.

During experimental testing, three variables are considered: accelerometer’s location, excitation direction, and accelerometer’s response direction. Left, middle, and right accelerometer’s locations are described above, while excitation direction and response direction are not considered in FEA as it computes an ideal system and thus yields identical results. Excitation direction describes the axis in which the RC is excited, while response direction denotes the data collected by the accelerometer. Excitation direction and response direction utilize the identical axes given in Figure 3a,b. Two common types of vibration are executed for comparison: free and random. Under each type of vibration, the RC component is attached to a rigid fixture represented by an aluminum block, which acts as an adapter between the system and the smart table or shaker. The block allows the RC to be bolted down, ensuring that fixture dynamics do not influence the RC. Since it is difficult to consistently displace such a stiff system by hand, free vibration is performed with a Modal Shop impact hammer where the RC component is excited in the desired direction and the time histories are collected in the *X*, *Y*, and *Z* responses. This process is repeated four more times to ensure consistency between tests. Excitations are performed in the *X*, *Y*, and *Z* axes with an impact hammer. Multiple impact locations are experimentally tested and those depicted in Figure 3 resulted in the least noisy data. All data are collected in Simcenter Testlab (Version 2019.1.3) and processed using MATLAB (Version R2022a).

**Figure 4:**
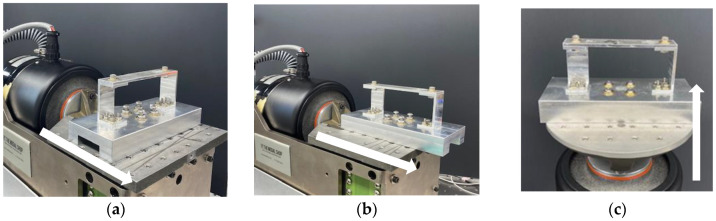
Shaker excitation in (**a**) *X*-axis, (**b**) *Y*-axis, and (**c**) *Z*-axis.

Random vibration is tested in a similar matter and attached to the same aluminum fixture, allowing the RC to be bolted to the shaker. Figure 4a–c illustrates the system during the *X*, *Y*, and *Z* excitations. Multiple excitation levels are tested to determine the minimum forcing with sufficient signal-to-noise ratio to accurately measure responses, while the maximum input is selected from the shaker’s operating capabilities corresponding to inputs of 1 × 10^−7^ V^2^/Hz and 1 × 10^−5^ V^2^/Hz, respectively. Outputs are compared through PSDs, which have units of g^2^/Hz, because they are the easiest to generate, provide the most intuitive plots, and have been used throughout the literature. Tests are conducted multiple times to verify repeatability and bolts are loosened and retorqued in accordance with Sandia’s challenge problem statement between tests for consistency. The structure is then modeled with and without an accelerometer to compare the experimental results to those extracted from the finite element simulations. They also provide initial insight on the impact that the accelerometers have on the system’s dynamics.

## 3. Finite Element Modeling: Effects of Accelerometer Location on the Modal Characteristics of the System

**Figure 5:**
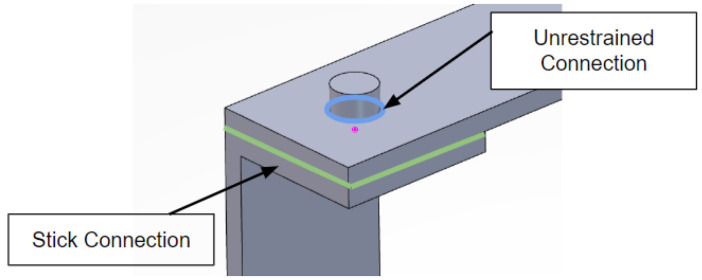
FEA stick and unrestrained constraints.

In this section, the results from the FEA are presented, in addition to the constraints used to provide a baseline for experiments. The results are generated using the ABAQUS solver in a modal analysis regime to guide the experiments. While FEA can be extremely insightful, it is important for simulation and laboratory conditions to match. Modeling bolted connections is notoriously difficult or computationally expensive [44,45,46,47] and typically requires experimental data to tune parameters due to the significant complexity of bolted joint systems. To approximate these nontrivial conditions, many such as Lacayo et al. [44] have used simplified constraints instead of modeling the bolts, nuts, and washers in preliminary models. In a similar manner, the authors have chosen stick and unrestrained constraints as two extreme cases to guide the experimental results. These two simplified extreme cases which are evaluated represent the high and low estimations that should bound the experiment. These two cases are achieved through stick and unrestrained constraints, which represent the high and low stiffnesses, respectively, that confine the results [44]. In the unrestrained configuration, only the bolt hole’s edges between the flat plate and the c-channels are in constant contact throughout the simulation. This edge is represented in blue in Figure 5. Only a small portion of each component is in contact so the structure moves almost freely, giving it the least restriction and serving as the lower frequency boundary in the most flexible scenario. Conversely, the stick constraint assumes the entire surface between the plate and the c-channels are coupled throughout the simulation, restricting any separation between the two. In Figure 5, the green lines indicate the plane of interaction. Because the surfaces move in unison, the simulation treats this structure as stiffer and thus yields a higher natural frequency.

**Figure 6:**
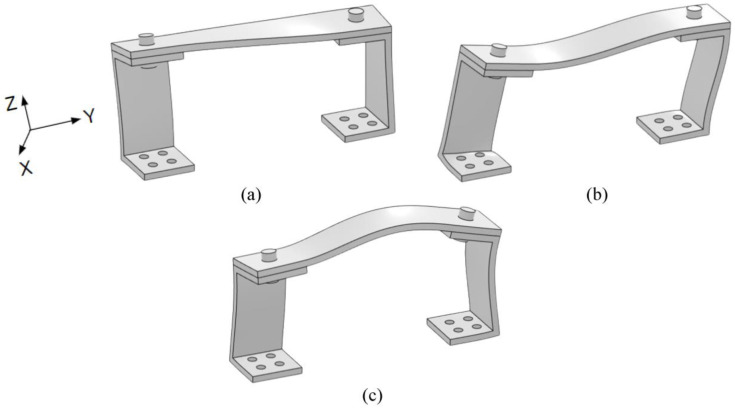
FEA picture for the (**a**) first, (**b**), second, and (**c**) third modes of the RC.

While both computational simulations are needed, the stick configuration represents a more realistic case for two reasons. First, the unrestrained simulations allow for the two components to freely penetrate each other in an unrealistic manner. Second, the torque from the bolts creates a conical pressure distribution across the touching surfaces that highly restricts movement between the two as if they are fixed together. Experimental results are expected to be between the two cases, but much closer to the stick configuration for these reasons. Mode shapes for the first three modes are identical between stick and unrestrained and are depicted in Figure 6.

The finite element results provide an understanding of the RC’s behavior to predict which accelerometer’s locations may have the most significant influence on the system’s dynamics and to target locations of low and high impacts. The first mode of vibration, for example, is highly torsional on the left side while the right side remains almost neutral throughout the simulation. It moves dominantly in the *X* and *Z* axes, with almost no movement occurring in the *Y*-axis or on the right side of the RC. It follows that placing an accelerometer on an area of large displacement like the left side can affect a much more significant decrease in natural frequency as the inclusion of an accelerometer adds additional mass which decreases the system’s natural frequency. Additionally, placement at any location other than a node decreases the structure’s stiffness when compared to the same simulation without an accelerometer. Similarly, accelerometer placement on the right side near a node may only cause a slight decrease in the natural frequency as it still adds mass to the system, but has a much smaller impact on the RC’s effective mass and the middle accelerometer placement will fall between the two. Moreover, it is expected that the accelerometers’ responses in the *X* and *Z* directions will be more beneficial to analyze as the displacement is more apparent than in the stable *Y* direction for the first mode of vibration.

Likewise, the second mode of vibration exhibits an in-plane rocking motion in the *Y*-axis that appears to have similar behavior for each accelerometer location in all three axes. As for the third mode of vibration, it has the largest displacement in the *Z* direction, followed by the *Y*-axis motion. Movement in the *X*-axis is limited in this mode. The right side experiences the most significant displacement while the left side has the smallest change, appearing that a node occurs at the left edge.

Finite element is also run with the inclusion of an accelerometer at each location and while the accelerometers have a large effect on the natural frequencies, the results indicate that the mode shapes did not change when compared to simulations without an accelerometer for the first three modes of the RC. Table 1 summarizes the FEA natural frequencies for the first three modes, with the no accelerometer condition used as the baseline.

When inspecting the stick and unrestrained natural frequencies for the RC with and without accelerometers, as expected, the stiffer stick condition produces higher natural frequencies than its unrestrained counterpart for each mode and accelerometer’s placement. The no accelerometer condition also shows the highest natural frequencies since it does not have the added sensor weight and natural frequency is indirectly proportional to mass. Analyzing the first mode of vibration, natural frequency increases as the accelerometer is moved from left to right. While the system’s mass is equal for each simulation with an accelerometer, its placement changes the effective mass of the RC and its coupling/influence on the dynamics of the system. Since the left side of the RC has the largest displacement in mode 1, the accelerometer at this location creates the greatest increase in the effective mass and the trend gradually decreases toward the right.

Looking at the second mode shape, motion is approximately uniform at each location across the top of the system. The natural frequencies in Table 1 support this finding as the left, middle, and right unrestrained frequencies are within 1% of each other. However, all three placements provide a noticeable decrease from the no accelerometer values implying that any accelerometer placement on the top of the RC will significantly skew the system’s unimpeded response. Concerning the third mode of vibration, its corresponding mode shape shows an anti-node or large displacement area between the middle and right accelerometers, but slightly closer to the middle. Thus, the middle accelerometer gives the lowest natural frequency due to the effective mass influence of the accelerometer, followed by the right and then the left, which is almost unaffected.

During experimental testing, the optimal accelerometer’s location is highly dependent on the vibratory mode of interest. FEA should first be run to determine the structure’s nodes and anti-nodes or largest displacement areas to target the nodes for sensor’s placement to minimize displacement of the accelerometers. The right accelerometer has the least effect on the displacement and effective mass for mode 1 and thus would be the best choice for accelerometer placement that aims to test the RC’s actual first natural frequency, as shown in Table 1. For the second mode, any placement along the top of the RC will have a noticeable impact on the system, suggesting that an accelerometer could instead be placed on the bottom side of one of the c-channels. Lastly, to collect the most accurate measurement of the third mode’s natural frequency, the left accelerometer’s placement is closest to the node and follows that it has no significant impact on the system. While FEA only provides insight of the natural frequencies and mode shapes, the accelerometer’s placement may also affect other dynamics of the system as well. Because the range of frequencies for the first three modes of vibrations can be between 350 Hz and 1300 Hz, the experimental testing is narrowed to only focus on the first mode of vibration in order to deeply investigate the effects of the accelerometer’s location on the linear and nonlinear characteristics of the RC system.

## 4. Experimental Results and Discussion

In this section, the experimental results performed in this study are shown and discussed for a range of frequencies near the fundamental mode of vibration. Free vibration testing is performed as an initial comparison to FEA findings. Using an impact hammer, natural frequencies and damping are calculated from time histories for the linear results/regime as t → ∞ and similarly for the nonlinear results/regime at t ≈ 0. These two extreme values may give an idea on the linear properties of the system and a trend for the nonlinear response of the system, respectively. Next, random vibration testing is carried out at multiple input levels to better characterize the system’s dynamics depending on the direction of excitation.

### 4.1. Free Vibration: Influence of the Accelerometer’s Placement on the First Mode of Vibration

#### 4.1.1. Frequency Analysis: Time Dependence of the Damped Frequency of the System

**Figure 7:**
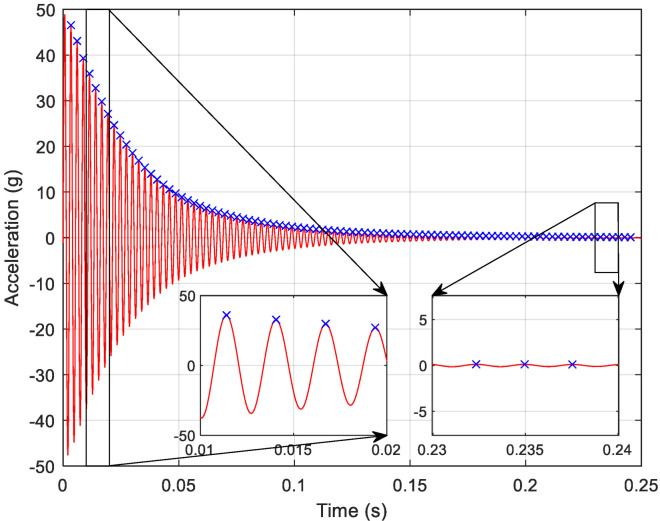
Time history for the left accelerometer *X*-excitation, *X*-response showing period change at the beginning and end of the impact.

**Figure 8:**
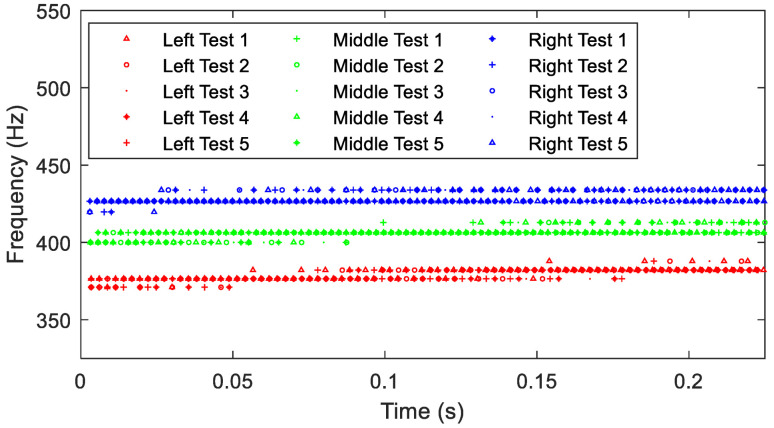
Variations of the fundamental damped frequency with respect to time for the three accelerometer’s locations when excited in the *X* direction and considering the *X* response.

The finite element results highlight the importance of the accelerometer’s placement from a computational perspective, but the same principles hold true on the experimental side. To determine the variation of the instantaneous damped frequency with respect to time during a free vibration test, time histories are collected. The plot presented in Figure 7 is for the left accelerometer when excited in the *X*-direction and its response is measured in the *X*-direction. After measuring the damped frequency at each period, the results show that the damped frequency increases with time from 400 Hz near impact to 413 Hz at the end of the test since the hammer imparts a high excitation on the system at t ≈ 0 with a gradual frequency increase as t → ∞ and the response dies out. In other words, the initial excitation causes nonlinear softening which decreases as the data are recorded further from impact toward a minimally excited system. There exists a slight fluctuation due to small uncertainty in the test data and limited frequency bins in the MATLAB code, as summarized in Figure 8. For free vibration testing, damped frequency measurements are almost identical regardless of *X*-, *Y*-, or *Z*-excitation. The damping ratio, however, is based on the displacement. During the free vibration testing, accelerometer placements that experience larger disturbances, such as the left and middle, have higher damping values because the system must travel a further distance before reaching equilibrium. *X*-excitations and responses produce higher amplitudes than *Y*-excitations and responses for the first mode and thus have higher damping values, with *Z*-excitations and responses falling between the two. This observation will be deeply discussed next.

Next, the time histories are used to calculate the fundamental damped frequencies. Results from the five tests for each accelerometer are given in Figure 8. Ranges for each test have excellent agreement, displaying the presence of multiple frequencies from impact to equilibrium. The right accelerometer’s response includes a concentration of data points at higher damped frequencies, suggesting a stiffer system that is more resistant to impact. The left accelerometer, however, has more data points at lower relative frequencies near t ≈ 0 and a broader response range. Placement of an accelerometer on the left side of the RC significantly increases the system’s effective mass compared to the right-side placement, as verified by FEA. The middle accelerometer data fall between the two.

**Figure 9:**
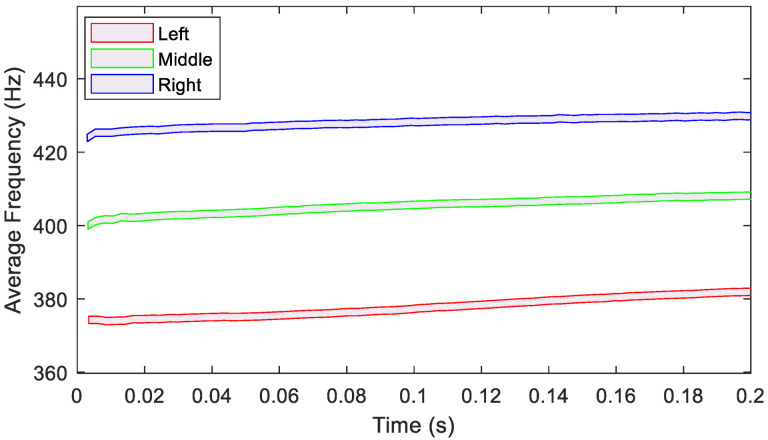
Average damped frequency variation with respect to time for accelerometers, *X* excitation, and *X* response considering 6 Hz uncertainty.

Due to the consistency between tests, responses are combined, and a running average is placed over the experimental data to reduce noise and demonstrate the trend in fundamental damped frequency change over time. From the previous plot, a 6 Hz uncertainty representing the range of possible frequencies for each time is considered for the accelerometers’ responses and the trends are plotted in Figure 9. Each curve starts at the lowest frequency at the beginning of the test that increases with time as the structure approaches equilibrium. It is important to note that the right and middle accelerometers have the smallest slope, while the right accelerometer experiences the quickest increase in frequency over time. This change is an effect of the damping for each condition, which is explained in the next section.

Data from the plots shown in Figure 8 and Figure 9 are collected in Table 2 and compared to the FEA results. As previously discussed, the FEA stick conditions more accurately represent the physical system as the free vibration results verify. Because the system behaves with a nonlinear softening response, the soft and stiff bounds are defined as the damped frequencies near t ≈ 0 and t → ∞, respectively. Since the system is excited to a consistent initial amplitude, t ≈ 0 corresponds to an initial amplitude of 50 g and t → ∞ represents a positive near-zero amplitude. For each accelerometer, stiff free vibration, which should greatly represent the linear damped frequency of the system, matches closely with the FEA stick condition, to approximately 1%. Both instances approximate a linear system, as is the nature of FEA, while the free vibration near equilibrium experiences only a small amplitude, making it a fitting linear approximation since the structure is minimally excited. The left accelerometer experiences the widest range between soft and stiff frequencies due to the softening added from the accelerometer’s placement at impact before approaching its linear regime. The middle and right accelerometers, however, impart a less significant influence on the system due to their placement closer to a node of the first mode. To this end, frequencies for these locations are easier to estimate by linear computational methods.

#### 4.1.2. Damping Characteristics and Dependence to Accelerometer’s Location

**Figure 10:**
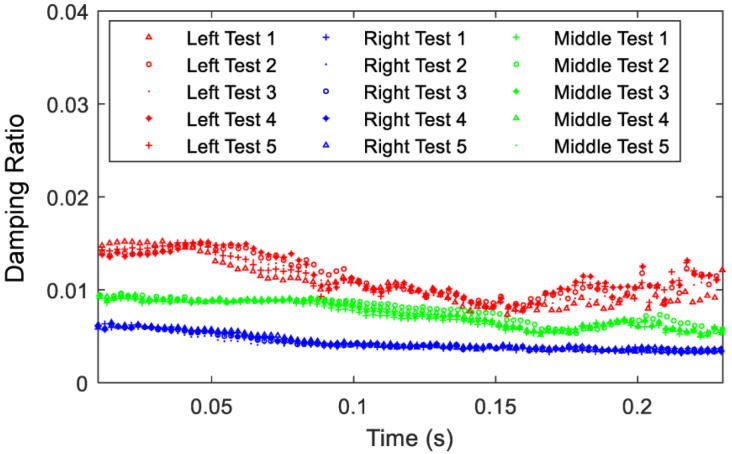
Variation of the instantaneous damping ratio with respect to time for the three accelerometers, *X*-excitation, and *X*-response.

Damping data are collected in a similar fashion from accelerometers’ time histories in Figure 10. It should be mentioned that the logarithmic decrement technique is employed to estimate the instantaneous damping ratio at each period. Results from each of the five tests display high repeatability, especially for the middle and right accelerometers. The left accelerometer’s data show a slight variability in damping ratio since this flexible configuration is the most sensitive to impact. Because excitation with the impact hammer is performed by hand, it is impossible to achieve the exact same force across all five tests, but the impacts are consistent at 50 g, and the trends are identical. While the RC is a stiff structure, it exhibits relatively higher damping ratios under higher excitations due to the large deformation and hence the possible activation of the nonlinearities in the system. Again, as t → ∞, the damping ratio approaches a constant value which should match the linear damping ratio of the system. It should be noted that the damping ratio values begin to fluctuate more under such low responses because the signal to noise ratio impacts the values at the culmination of the test.

**Figure 11:**
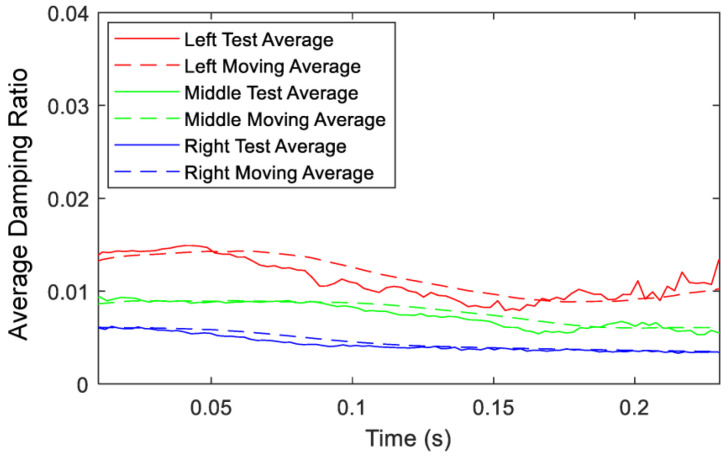
Average damping ratio variation with respect to time for the five tests for the three accelerometers, *X*-excitation, and *X*-response.

Since it is shown that each test has high repeatability, the responses are combined, and averages are placed over the experimental data to demonstrate the trend in damping ratio in Figure 11. The test average is an average of the five curves, while the moving average is a simple filter for the data noise that averages each new point with a previous number of data points to better illustrate the trend. From an instantaneous damping ratio perspective, the left accelerometer creates the highest damped system for the entire time history because it undergoes more displacement than the other accelerometers and due to the influence of its mass on the effective properties of the system and its fundamental natural frequency. However, all accelerometers experience nonlinear variation in the instantaneous damping ratio with respect to time, even the right accelerometer location which was demonstrated to be nearly linear and has minimal contribution to the RC’s dynamics. This finding demonstrates that the RC itself is a nonlinear system, which is a reason for its introduction to the literature as a benchmark system of interest. By placing an accelerometer at high displacement locations, the added instrumentation may affect a profound change in the RC’s inherent responses from both damping and frequency perspectives.

To provide further insight of the RC’s damping ratio, the minimum and maximum values are denoted in Table 3. Instantaneous damping ratio values from the beginning to end of the test drop between 37.9% for the left accelerometer and 45.8% for the right accelerometer. Accelerometer placements that create a more flexible system yield higher overall damping in the system and display a less of a drop during the test, from a damping ratio perspective. Similarly, as the accelerometer moves from left to right closer to the node of the first mode, damping ratio drastically decreases. Comparing the most extreme cases, damping at t ≈ 0 for the left accelerometer is over double that of the right accelerometer and the damping ratio as t → ∞ is nearly triple. The same trend exists on a smaller scale for the middle accelerometer when compared to the right. Free vibration results conclude that placement of an accelerometer further from the node causes a significant increase in damping ratio and a significant decrease in the natural frequency for the first mode of vibration of the RC. It should be mentioned that the effective damping in the RC system will also be changeable with respect to time and hence the presence of nonlinear damping for this system.

### 4.2. Random Vibration: Effects of the Accelerometer’s Location on the System’s Dynamics

Next, random vibration testing is performed as a comparison to free vibration and FEA. Random vibration allows for easier testing at multiple excitation values to gain a deeper understanding of the nonlinear system’s dynamics while exciting all modes, showing a possible presence of subharmonics in the data. Left, middle, and right accelerometers are compared in addition to the excitation direction and response axis from each accelerometer. The same fixture from free vibration is used for consistency. It should be noted that the experiment was purposefully set up and intentionally tuned so that any areas of noise remain outside regions of resonance. This design in collecting data allows the findings to be plotted without filtering, preserving the raw data in an unaltered form.

#### 4.2.1. Effects of the Accelerometer’s Location on the Resonant Frequencies

##### Right Accelerometer

**Figure 12:**
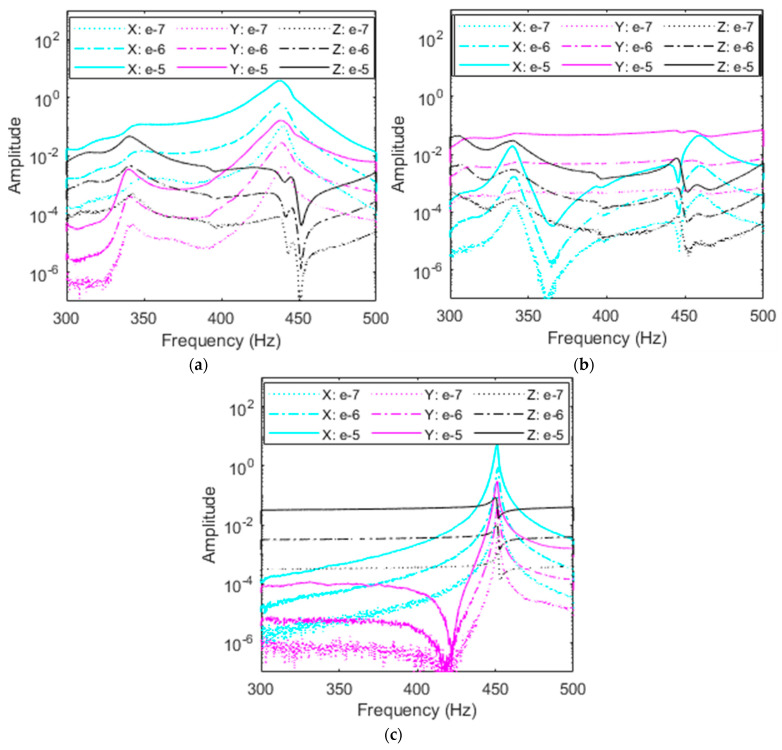
Amplitude comparison in g^2^/Hz of response direction among (**a**) *X*-, (**b**) *Y*-, and (**c**) *Z*-excitations for the right accelerometer and each response direction at three levels of excitation.

As demonstrated by FEA and free vibration, the right accelerometer adds the least influence on the RC’s dynamics and provides the closest response to no-accelerometer simulations. Data from the *X*-, *Y*-, and *Z*-excitations are portrayed in Figure 12a–c, respectively, with the responses in all three axes in each plot. It is important to note that no filtering is used in the remaining plots to preserve the data. While some portions of the plots are noisy, these regions do not interfere with the natural frequencies and thus are not of concern. For all excitations, resonant peaks appear in the range of 440–460 Hz, corresponding to the first mode of vibration. There exists a small fluctuation in these peaks due to the influence of subharmonics from the second mode as this peak is also approximately half the value of the second natural frequency, as shown in Table 1. It is also important to note the amplitudes of each plot; the *X*- and *Z*-excitations reach the highest amplitude of over 10, while the *Y*-excitation amplitude is two orders of magnitude lower just under 0.1.

From the FEA first mode shape, the RC displaces the most in the *X* and *Z* axes with little motion in the *Y*-axis which matches the amplitudes of the plots. Similarly, it is expected that the response axes follow a similar trend. A second set of peaks are present near 340 Hz in the *X* and *Y* axes excitations, likely due to subharmonics from the second and/or third modes. These peaks are not active during classical *Z*-excitation. Excitations ranging from 10^−7^ V^2^/Hz to 10^−5^ V^2^/Hz show that PSDs for the right accelerometer experience minimal nonlinear softening, meaning that as excitation is increased, the identified natural frequencies remain at nearly the same values. This confirms the results obtained from free vibration testing. In fact, the right accelerometer had a negligible effect on the variations of the fundamental damped frequency with respect to time, as shown in Figure 9 and Table 2. It should be mentioned that this small change in the fundamental resonant frequency is clearly seen when the RC is excited in the *X*-direction and the response is measured in the same direction, as shown in Figure 12a. Indeed, a nonlinear softening behavior is observed when the excitation input level is increased.

##### Middle Accelerometer

**Figure 13:**
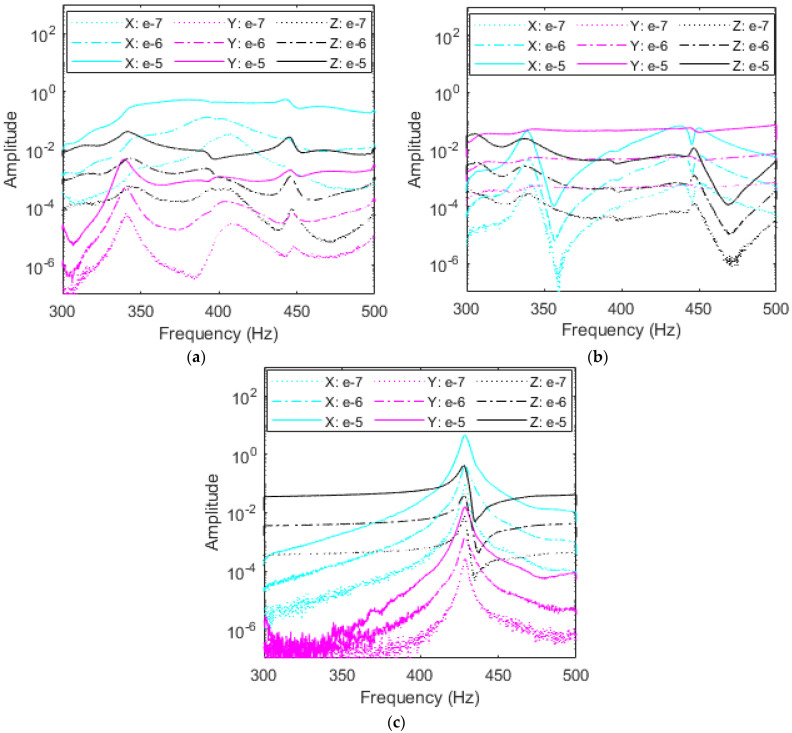
Amplitude comparison in g^2^/Hz of magnitude of each response direction among (**a**) *X*-, (**b**) *Y*-, and (**c**) *Z*-excitations for the middle accelerometer.

Investigating the impacts of the accelerometer’s placement on the system’s dynamics for various levels of excitations and considering various excitation directions, Figure 13a–c shows this investigation for the middle accelerometer. It follows from the random vibration results plotted PSDs in Figure 13 that the resonance region shifts when the accelerometer creates a softer system. This is expected from the finite element results and free vibration testing for the first mode of vibration. Indeed, resonant peaks near 430 Hz for the *Z*-excitation match with FEA and free vibration results, as shown in Table 2. It should be mentioned that some discrepancies may take place between free and random vibrations results due to the kind of excitation and experimental setup. Additional resonant peaks are still present near 340 Hz and 440–450 Hz from higher modes’ subharmonics, but have shifted toward the right by a few Hz, combining with another set of smaller peaks. Because these secondary peaks are subharmonics, they do not move by the same frequency as the resonant peak as they appear in 1/n fractions of higher modes which may cause them to overlap and experience coupling.

Inspecting the plots in Figure 13a,c, the amplitudes demonstrate that *X* and *Z* excitations provide the most beneficial results and have the largest displacement in the first mode, as shown for the right accelerometer’s data. Interestingly, *X*-excitation yields a nonlinear softening effect in the *X* and *Y* responses as excitation input is increased from 10^−7^ V^2^/Hz to 10^−5^ V^2^/Hz. As the accelerometer moves away from the node present of the right side of the RC, the accelerometer’s mass loading makes the bolted joint nonlinearities more pronounced. This effect leads to nonlinear softening in the system, either decreasing resonant frequency and or creating the broadband region that Figure 13a shows as curves become flatter with increased *X* excitation as the resonant frequency at 405 Hz decreases in all directions with the most significant change of 25 Hz for the *X* response in cyan.

##### Left Accelerometer

**Figure 14:**
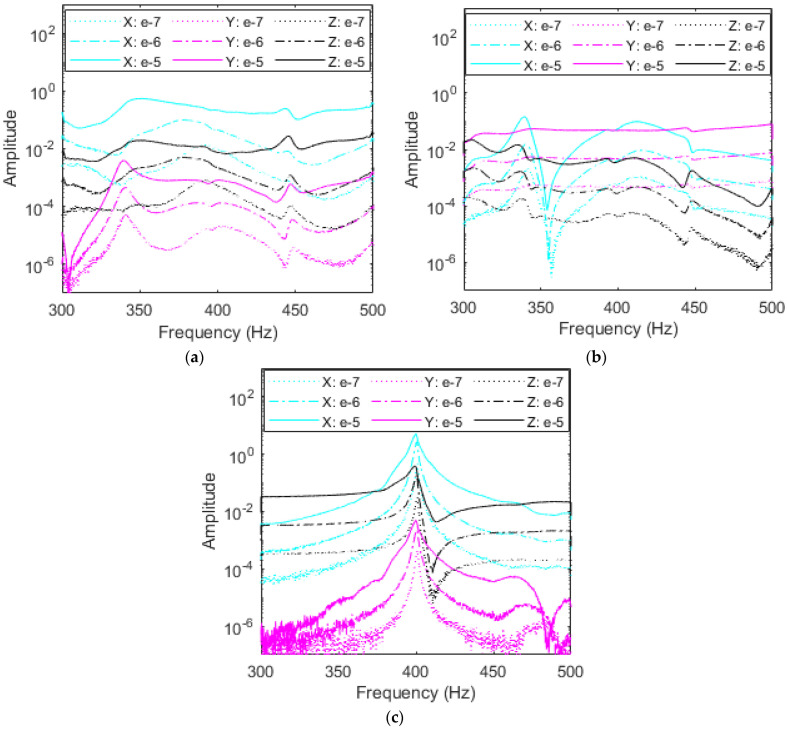
Amplitude comparison in g^2^/Hz of magnitude of each response direction among (**a**) *X-*, (**b**) *Y-*, and (**c**) *Z*-excitations for the left accelerometer. The backbone curves on *X*-excitation are in red.

Evaluating the influence of the left accelerometer on the dynamics and characteristics of the RC system is important for the first mode of vibration based on the finite element and free vibration results due to the significant reduction in the system’s natural frequency, increase in damping, and obvious presence of nonlinear softening. It follows from the plots in Figure 14a–c that the left accelerometer once more experiences a resonant frequency shift due to mass loading. For the left accelerometer, high amplitudes of *X-* and *Z*-excitation compared to *Y*-excitation match displacement quantified by mode shapes. Excitations in the *Y*-axis fail to excite the first mode of vibration because the first mode displacement is nearly constrained to the *X*-*Z* plane and elicits heavy subharmonic influence from rocking in the *Y*-axis of the second and third modes of the RC system. The *Y*-excitation is more susceptible to subharmonics due to the motion of the second and third modes. Classical *Z*-excitation peaks center at 400 Hz with a pure linear regime, while the *X*-excitation exhibits significant nonlinear softening. As the input excitation value is increased in the *X*-direction, as shown in Figure 14a, *X* and *Z* resonant frequencies decrease by almost 50 Hz as outlined by the red backbone curves. The shift here demonstrates that the left accelerometer placement contributes highly nonlinear effects to the RC and is most sensitive to input excitation. As in the middle and right accelerometers’ plots, subharmonic peaks due to higher modes also remain at similar frequencies of 350 Hz and to 450 Hz.

#### 4.2.2. Dependence of Nonlinear Damping on Accelerometer’s Placement

**Figure 15:**
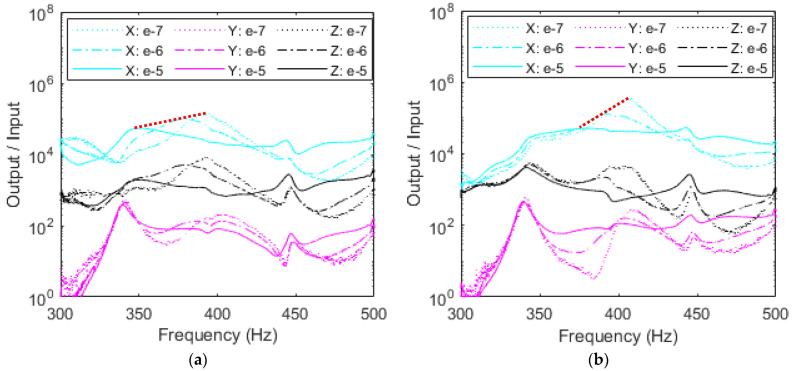

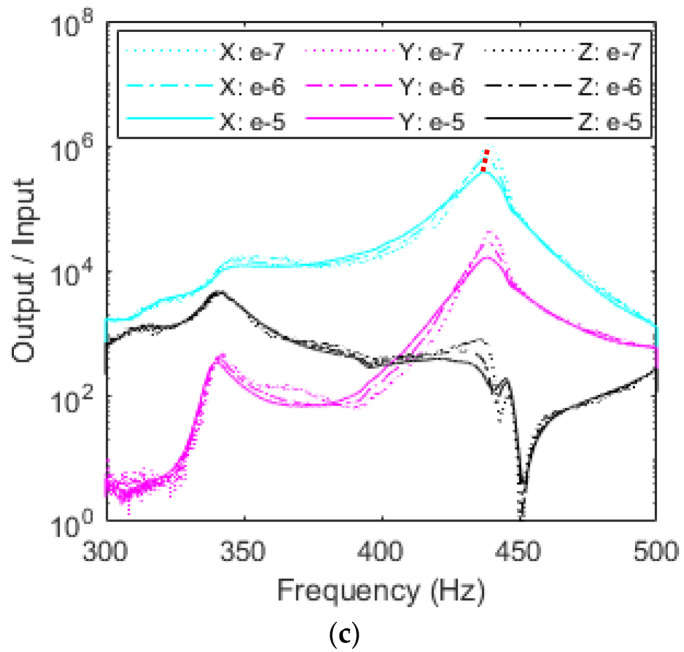
Comparison of magnitude of each response direction among (**a**) left, (**b**) middle, and (**c**) right accelerometers and input level in *X*-excitation. The backbone curves are in red.

Based on the free vibration results shown in Figure 11 and Table 3, it was proved that as the accelerometer is shifted from right to left, the system exhibits more nonlinearities due to the damping. To further investigate this phenomenon, *X*-excitation, which contributes the most nonlinearities to the RC, output over input is plotted in Figure 15a–c for all accelerometers. Inspecting these plots, it is clear that the increase of the input forcing is accompanied by a decrease in the output over input amplitude which indicates the presence of nonlinear damping for all three accelerometers. This is in full agreement with the results shown in Figure 11 from the free vibration testing.

For the left accelerometer, resonant frequency shifts the largest amount as excitation is increased, the right accelerometer frequency shifts just a couple Hz, while the middle accelerometer lies between the two. This trend is illustrated by the red dotted backbone line and suggests that there is a high dependence on accelerometer location to nonlinear damping in the system. Since the *X*-responses exhibit the largest amplitude, they similarly experience the most nonlinear softening in testing and provide the most valuable insight into the RC’s nonlinearities. Further analyzing the response of the left accelerometer shows more interesting results in Figure 15a. It was previously discussed from the FEA and free vibration that the *X*-response shows the largest amplitude when compared to the other axes, for the first mode of vibration. It follows then that the *X*-response, which is most prone to change, also sees a prominent nonlinear softening. The 50 Hz change over the backbone curve represents an 8% variation which would only increase further under larger inputs. The *Z*-response demonstrates similar behavior in that the left accelerometer is highly prone to shaker level. This backbone curve exists only in *X*-excitation from the shaker for *X*- and *Z*-responses from the accelerometers. Like Figure 14, peaks at 450 Hz are likely a subharmonic of the second mode of approximately 900 Hz.

The middle accelerometer exhibits similar behavior with nonlinear softening for the *X*-response, and an almost identical *Y*-response, but the *Z*-response peaks occur at almost the same frequency without clear evidence of nonlinear softening due to the possible coupling with the subharmonics of higher modes. The right accelerometer shows no nonlinear softening regardless of response direction, implying that as the accelerometer is placed closer to the anti-node or large displacement area, it adds nonlinear softening into the system which would otherwise not exist.

#### 4.2.3. Comparison of Accelerometer’s Location on the Resonant Frequencies of the RC System

**Figure 16:**
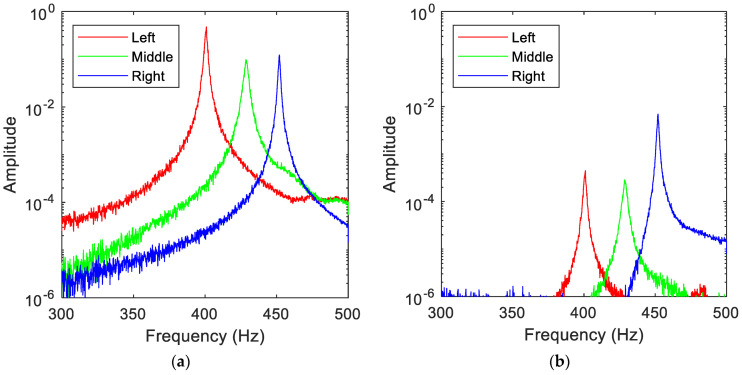

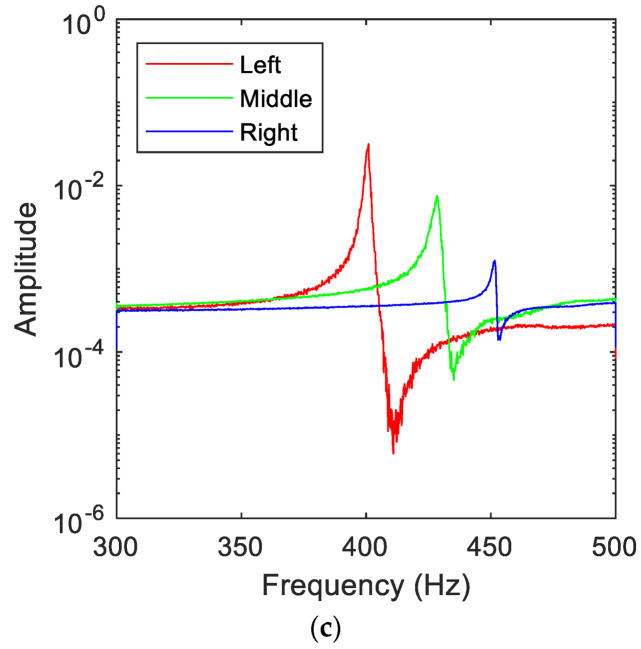
Comparison of (**a**) *X-*, (**b**) *Y-*, and (**c**) *Z*-responses for each accelerometer *Z*-excitation at 1 × 10^−7^ V^2^/Hz.

For each accelerometer location, *Z*-excitation is chosen in order to estimate the resonant fundamental frequency since the RC system behaves more linearly and avoids subharmonics based on previous graphs. This selection results in the cleanest responses for each accelerometer given in Figure 16. The smallest excitation closest to the linear regime of 1 × 10^−7^ V^2^/Hz is plotted, but resonant fundamental frequency remains almost identical at 451 Hz regardless of shaker input for 1 × 10^−5^ V^2^/Hz through 1 × 10^−7^ V^2^/Hz. *Z*-excitation has the cleanest responses because all bolted joints on the RC occur at interfaces between the top plate or the aluminum fixture and c-channels. Each contact surface lies solely in the *X*-*Y* plane, meaning that forcing in the *Z*-axis is axial to this plane and the bolts. Therefore, slip between washers, bolts, and components is minimized.

The PSDs demonstrate that the RC’s resonant frequency is highly dependent on the placement of accelerometers. As validated by FEA and free vibration testing, the left accelerometer causes the lowest natural frequency for the first mode of vibration, followed by the middle and right accelerometers. Responses in the *X*-direction show the highest amplitude as the first mode is torsional with the largest deflection in this axis. We can also expect that the *Y*-direction amplitudes are an order of magnitude less because resonance demonstrates minimal movement here with most displacement occurring in the *X* and *Z* axes. It is also interesting to note the small peak near 460 Hz in the PSDs for each accelerometer. The second natural frequency for all accelerometer’s locations is just over 900 Hz, meaning this peak occurs at approximately half the second mode suggesting the possible presence of a subharmonic response. All responses match with FEA mode shapes and free vibration data.

Overall, FEA, free vibration, and random vibration data show excellent agreement. Table 4 summarizes the maximum and minimum frequencies across all excitation axes and directions. For each accelerometer location, stick configuration, which closely represents laboratory conditions, is compared to upper and lower boundaries from free and random vibration across each excitation and response direction. The soft RC condition for free vibration should be closer to high impact at t ≈ 0 and to high excitation for 1 × 10^−5^ V^2^/Hz for random vibration. The hard condition for free vibration corresponds to low impact as t → ∞ and to low excitation for 1 × 10^−7^ V^2^/Hz for random vibration. Soft and hard excitations give important insights on frequency boundaries for the RC system’s first mode of vibration. It is important to note that the stiff free and random vibration values have excellent agreement with FEA results. While FEA is not an exact determination of natural frequencies, it is a great baseline to verify test data. Free and random results correspond within 2%. Under free vibration, the frequency change between soft and stiff conditions is nearly identical, ranging only from 12 to 16 Hz as it is difficult to impart a large force with the hammer.

Looking at the random resonant frequencies, there is a wider gap between soft and stiff excitations notably for the left accelerometer. Because the left accelerometer is in a location of such high displacement for the first mode, changes in input excitation cause much wider variation since the system experiences a large increase in effective mass. This means placing an accelerometer on the left side of the RC will drastically change its dynamical responses due to the large displacement area present there in the first mode.

## 5. Conclusions

Some form of instrumentation is required to measure resonant frequencies and characterize the dynamics of any system. Due to their simplicity and cost, accelerometers are often chosen for this purpose and can provide reliable results if placed correctly. While placement exactly at the node of a system would have a prohibitively low signal-to-noise ratio, a slight offset ensures that the added mass to a system from accelerometers does not significantly skew the dynamics of the system being measured. Placing the accelerometer on the far-right side minimizes mass loading and leads to the smallest effect on the removable component’s natural frequencies and damping. Intuitively, this result is expected as the right accelerometer experiences the shortest displacement at the first mode of vibration. Thus, accelerometers should be near nodes on the structure as they have the least change in position. However, nodes change and are dependent on which of the structure’s natural frequencies are excited. If ideal placement near a node of the RC is not possible, using an accelerometer may still yield helpful results under one of two conditions. First, the RC is excited vertically (*Z*-direction in this study) to ensure that the surfaces and bolts in contact do not undergo shear forcing. Responses in any axis can accurately measure the RC’s dynamics independent of the value of input forcing from the shaker. Alternatively, the RC should be minimally excited in the *X*-direction to avoid *Y*-excitation for the RC’s first mode of vibration.

## Figures and Tables

**Table 1:** Comparison of stick (S) and unrestrained (U) natural frequencies (Hz) for accelerometer placement for the first three modes of vibration.

	No Accelerometer		Left		Middle		Right	
Mode	S	U	S	U	S	U	S	U
1	431.1	385.4	383.5	341.0	413.3	363.2	427.7	379.2
2	971.6	752.0	906.9	703.5	914.9	695.9	916.1	688.8
3	1262.8	893.2	1255.5	831.0	1100.0	769.1	1124.8	831.1

**Table 2:** Comparison of accelerometer frequency ranges in Hz of FEA unrestrained (U) and stick (S) with free vibration for t ≈ 0 (soft) and t → ∞ (stiff).

	Left		Middle		Right	
**FEA**	**U**	**S**	**U**	**S**	**U**	**S**
	341.1	383.5	363.2	413.3	379.2	427.7
**Free**	**Soft**	**Stiff**	**Soft**	**Stiff**	**Soft**	**Stiff**
	371.0–376.5	382.1–387.9	400.0–406.3	406.3–412.9	419.7–426.7	426.7–433.9

**Table 3:** Damping of accelerometers for t ≈ 0 (soft) and t → ∞ (stiff).

Location	t ≈ 0	t → ∞	Percent Change
Left	0.014738	0.0091540	−37.9%
Middle	0.009329	0.005427	−41.8%
Right	0.006378	0.003458	−45.8%

**Table 4:** Comparison of FEA, free, and random vibration natural frequency in Hz, with minimum and maximum values for soft and stiff conditions across all excitation axes and directions.

	Left		Middle		Right	
	U	S	U	S	U	S
**FEA**	341.1	383.5	363.2	413.3	379.2	427.7
	**Min**	**Max**	**Min**	**Max**	**Min**	**Max**
**Free**	371.0	387.9	400.0	412.9	419.7	433.9
**Random**	351.0	392.4	380.0	407.8	437.9	439.6

## Data Availability

Data will be made available on reasonable request.
